# Phytocomplex Influences Antimicrobial and Health Properties of Concentrated Glycerine Macerates

**DOI:** 10.3390/antibiotics9120858

**Published:** 2020-12-01

**Authors:** Maura Di Vito, Margherita Gentile, Paola Mattarelli, Lorenzo Barbanti, Laura Micheli, Claudia Mazzuca, Stefania Garzoli, Mattia Titubante, Alberto Vitali, Margherita Cacaci, Maurizio Sanguinetti, Francesca Bugli

**Affiliations:** 1 Dipartimento di Scienze e Tecnologie Agro-Alimentari, Università di Bologna, Viale G. Fanin 42, 40127 Bologna, Italy; paola.mattarelli@unibo.it (P.M.); lorenzo.barbanti@unibo.it (L.B.); 2 Dipartimento di Scienze di Laboratorio e Infettivologiche, Fondazione Policlinico Universitario A. Gemelli IRCCS, Largo A. Gemelli 8, 00168 Rome, Italy; margherita.gentile01@unicatt.it (M.G.); margherita.cacaci01@icatt.it (M.C.); maurizio.sanguinetti@unicatt.it (M.S.); francesca.bugli@unicatt.it (F.B.); 3 Dipartimento di Scienze e Tecnologie Chimiche, Università degli Studi di Roma ‘Tor Vergata’, V.le della Ricerca Scientifica 1, 00133 Rome, Italy; laura.micheli@uniroma2.it (L.M.); claudia.mazzuca@uniroma2.it (C.M.); mattia.titubante@gmail.com (M.T.); 4 Dipartimento di Chimica e Tecnologie del Farmaco, Università di Roma “Sapienza”, Piazzale Aldo Moro 5, 00100 Rome, Italy; stefania.garzoli@uniroma1.it; 5 Istituto di Scienze e Tecnologie Chimiche “G. Natta”—C.N.R., Largo F. Vito 1, 00168 Rome, Italy; alberto.vitali@scitec.cnr.it; 6 Dipartimento di Scienze Biotecnologiche di Base, Cliniche Intensivologiche e Perioperatorie, Università Cattolica del Sacro Cuore, Largo A. Gemelli 8, 00168 Rome, Italy

**Keywords:** *Alnus glutinosa*, *Carpinus betulus*, *Ficus carica*, IM-SPME/GC-MS system, *Ribes nigrum*

## Abstract

The purpose of this study was to correlate the chemical composition of four commercial concentrated glycerine macerates (C-GMs), produced through the same extraction method, with their in vitro antimicrobial, antioxidant, and immunomodulatory properties, in order to evaluate their potential for healing upper airway diseases. C-GMs of *Carpinus betulus* (CB), *Ficus carica* (FC), *Alnus glutinosa* (AG) and *Ribes nigrum* (RN) were studied. The quality was evaluated using HPLC and IM-SPME/GC-MS systems; anti-oxidant and anti-microbial activities were assessed by the respective DPPH test, and micro-broth dilution test performed against 10 strains of *Streptococcus pyogenes* and 10 probiotic strains. ELISA and MTT tests were used to assess the immunomodulatory activity and the cytotoxicity of C-GMs, respectively. A significant correlation was found between the number of active compounds and the in vitro C-GMs effectiveness. Furthermore, the C-GMs of AG showed the best anti-microbial activity on pathological strains and, together with CB, the best anti-oxidant activity. The ELISA test exhibited a good immunomodulatory activity of RN. In vitro data support the integrated use of C-GMs of CB, AG, and RN in presence of airway diseases, and highlight the importance of standard procedures in cultivation, harvest and post-harvest treatments, as a premise for C-GMs with consistent characteristics.

## 1. Introduction

Gemmotherapy is a young branch of phytotherapy developed in the second half of the 20th century. It was established by Henry (1959) [1] as herbal medicine and bases its principles on the therapeutic potential of meristematic tissues of plants. Henry developed a method to extract the active ingredients from the buds using cold maceration in water, alcohol, and glycerol to obtain products named glycerine macerates (GMs). His studies laid the foundations of the modern gemmotherapy [2] or meristemotherapy. Subsequently, several French and Italian scientists developed and perfected Henry’s studies [3,4]. In 1965, the eighth French pharmacopoeia described [5] for the first time the preparation of GMs, which were then reported in the subsequent Italian pharmacopoeias [6]. According to pharmacopoeias, GMs are obtained immediately after harvest through the maceration of different fresh meristematic tissues [7]. In these guidelines, it is reported that before their use, GMs need to be diluted at the first Hahnemannian dilution (1DH) according to homeopathic procedures. Recently, the use of concentrated GMs (C-GMs), which are undiluted to 1DH, has been spreading. 

These C-GMs are natural extracts that lose their homeopathic characteristics and acquire those of traditional herbal medicine with more concentrated and easily detectable active ingredients [3,8]. C-GMs are about 10 times more concentrated than GMs, and therefore are used at lower doses (adults: about 5–15 gtt/day) than those used for diluted extracts (adults: up to 1 gtt/kg/day). As known, the most critical aspect of formulation based on natural substances is the chemical variability dependent on both the cultivation practice and the extraction process used to produce these extracts. Two are the extraction methods most commonly used. The first involves maceration of fresh buds in an *ana parti* solution of water, alcohol and glycerol, while the second involves maceration in an equal volume of alcohol and glycerol. In the first method, water and alcohol participate in the extraction of hydrophilic active components, and the final products have a lower alcohol and glycerol concentration than the extracts obtained with the second method in which the only water present is that contained in the meristematic tissues. To date, very few studies have evaluated the quality and effectiveness of GMs [9,10,11], and no study has been developed on C-GMs. These kinds of products are already on sale, and their potential for healing a series of diseases is claimed based on clinical evidence, although no relationship has been established between their effects, on one side, and their composition and properties on the other.

The aim of this study was to correlate the chemical composition of four commercial C-GMs extracted from different plant species (*Carpinus betulus* L., *Ficus carica* L., *Alnus glutinosa* (L.) Gaertn and *Ribes nigrum* L.), three of which are traditionally used in presence of upper respiratory tract diseases [2], with some relevant properties in the treatment of airway diseases, such as anti-oxidant, anti-microbial, and immunomodulatory properties. To achieve this goal, two commercial C-GMs for each species, obtained by the same extraction method, have been focused.

## 2. Results

### 2.1. HPLC Analysis

The HPLC analysis performed on the *F. carica* (FC) samples (Table 1) demonstrates that FC-B is characterized by a higher concentration of myricetin (t = 2.7 min), while FC-A has a higher signal related to quercetin, or better, to its degradation product at t = 6.3 min.

**Figure 1:**
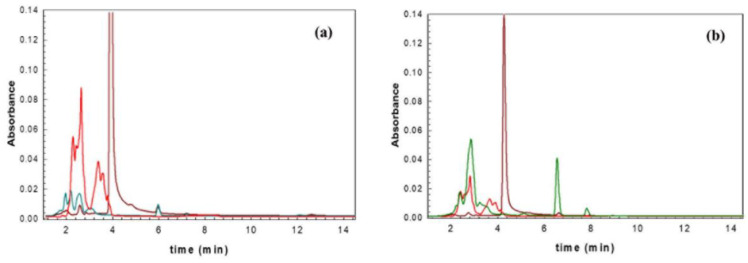
Superposition of quercetin (brown) and myricetin (red) signals with those of FC-B (**a**) and FC-A (**b**) samples.

Comparing the profile of each C-GM with both myricetin and quercetin standards, FC-A appears to be characterized by the typical signal of myricetin at t = 2.7 min, and a small amount of quercetin is identified at t = 4 min and 6.3 min (Figure 1a). In the FC-B sample, myricetin can be easily identified, and only the signal related to the by-product of degradation of quercetin (Figure 1b) can be detected. The signals at t = 6.3 min were assigned to the oxidized quercetin or to a compound deriving from its degradation. The analysis performed on the C-GMs of *C. betulus* (CB) shows a similar amount of myricetin and the absence of quercetin in all the tested samples. *A. glutinosa* (AG) analysis shows that myricetin and quercetin are more concentrated in AG-B than AG-A. Similarly, the *R. nigrum* (RN) samples show that RN-B has values of myricetin 3 times higher than RN-A. 

### 2.2. GC-MS Analysis

With IM-SPME/GC-MS, 7 volatile constituents and 3 three fatty acids were identified, which are listed in Table 2. Only in RN-A and RN-B samples the following components were found, at the respective contents: terpinolene (8.5%, 15.8%), terpinen-4-ol (33.1%, 33.5%), α-methylbenzenepropanamine (13.2%, 11.6%), spathulenol (15.3%, 15.9%) and caryophyllene oxide (29.8%, 31.1%). Octanoic acid was found only in FC-B sample (9.9%), while dodecanal only in AG-B sample (3.3%). FC-A and FC-B, CB-B and AG-B samples were rich in fatty acids. In particular, palmitic acid was the main component in FC-A (64.8%) followed by linoleic acid (22.8%) and linolenic acid (12.3%). On the contrary, in FC-B, the quantities of the 3 fatty acids were distributed more evenly (31.7%, 29.3%, and 29.0%, respectively). In the respective CB-B and AG-B samples, palmitic acid (20.5% and 31.9%), linoleic acid (28.0% and 29.4%), and linolenic acid (51.4% and 35.4%) were retrieved, while no compound was detected in both CB-A and AG-A samples. In conclusion, the findings clearly show that B-C-GMs were more concentrated with respect to A-C-GMs.

### 2.3. Glucose Analysis

Results obtained through the calibration curve (y = 0.440x − 0.00233; R^2^ = 0.997) are reported (Table 3). The glucose concentration in AG samples was very low, in one case (AG-B) under the detection limit of the biosensor. The matrix effect was observed for FC-B. 

### 2.4. DPPH Test

The DPPH test was performed to investigate the antioxidant activity of the C-GMs. Both C-GMs and producers were shown to be significant at the ANOVA. In the former case, the statistical relationship IC_50_-CB < IC_50_-AG < IC_50_-RN <IC_50_-FC was demonstrated in the average of the two producers. In the latter case, the statistical relationship IC_50_-B < IC-_50_-f was proved true in the average of the four C-GMs. However, the significant C-GMs × producers interaction (Table 4) indicates quite comparable IC_50_ values for two C-GMs (CB and AG) across the two producers, while diverging IC_50_ values were shown for the other two C-GMs (FC and RN). In the latter case, much lower concentrations were needed to attain the IC_50_ with the B-C-GMs vs. A-C-GMs.

### 2.5. Antimicrobial Activity

C-GMs from the two sources have similar effectiveness against all microbial strains tested, and the AG the main antimicrobial action (Table 5). Furthermore, the inhibitory activity occurs at dilutions at least twice lower than the dilutions needed for cytocidal activity. MIC90 and MBC90 of *S. pyogenes*, showing both the MIC and MBC of the different probiotic strains, and the relative values of IC90 e BC90 have been described (Table 5). Data show that probiotic strains are more resistant than pathogenic strains, and that AG has the best profile because at concentrations ≤ 2.5% *v*/*v* it selectively inhibits pathogenic strains.

### 2.6. ELISA Test

The OD values of cytokines and the ratio between the pro-inflammatory cytokines and IL-13, the only cytokine that has exclusively anti-inflammatory activity, are reported in Table 6. IL-1β, IL6, and IL-8 were not included in this table because their signal was too high, i.e., out of range with respect to the indications provided by the manufacturer. PBMCs stimulated with LPS + C-GM of RN-B show a value of the ratio similar to that of untreated PBMCs, whereas, the cells treated with LPS + C-GM of RN-A exhibit a value of the ratio significantly higher than that of PBMC (*p* < 0.05).

### 2.7. Cytotoxicity Test

**Figure 2:**
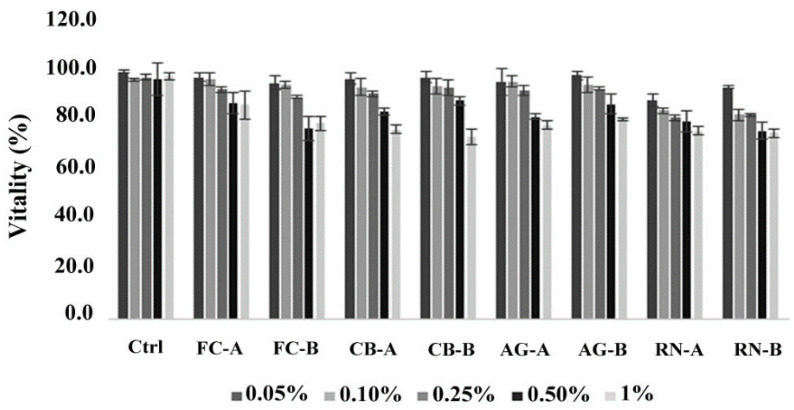
Cytotoxicity assays performed with a Cell Titer Blue assay on 3T3 fibroblasts at 24 h after cell synchronization. The error bar indicates the standard deviation.

The cytotoxic action of C-GMs is not significantly harmful at the concentration tested (Figure 2). This result is comparable among C-GM species and dose-dependent. In particular, concentrations lower than or equal to that obtained by dissolving 5 drops in half a glass of water (about 250 µL in 100 mL correspond to 0.25% *v*/*v*) do not show cytotoxic effects. However, a moderate decrease in vitality is observed at concentrations equal to 0.5% and 1% *v*/*v*. 

## 3. Discussion

Henry and his successors stated that the maceration of the meristematic tissues should take place in a primary solution characterized by alcohol-water-glycerol in equal parts (each at 33% *v*/*v*), establishing a protocol suitable to simultaneously extract alkaloids, hydrophilic, and hydrophobic compounds from meristematic tissues [12,13,14]. This protocol differs from that published in Pharmacopoeias, in which a binary extraction method characterized by a primary solution made with 50% alcohol and 50% glycerol is described [5,15]. It may be evinced that the two protocols generate different products in terms of chemical composition and efficacy. However, even products extracted with the same method may have a chemical variability due to the processing method or plant intrinsic variability. In our study, we decided to analyze C-GMs obtained according to the Henry method characterized by a 10-time lower alcohol content than in the other C-GMs, because these natural products are also recommended in pediatrics, and a lower alcohol concentration is desirable. A preliminary investigation on the Italian market was carried out, and six producers of C-MGs were identified. Two of them apply the extractive method described in the European Pharmacopoeia, two were described by Henry [1,2], and the last two do not indicate the method used. Three C-GMs, traditionally indicated as elective integrated therapies in the treatment of colds, rhinitis, sinusitis, pharyngitis and bronchitis, were chosen for the study. In particular, CB is used as antispasmodic and antitussive [12,15], RN as the major anti-inflammatory macerate [16,17,18], and AG as the complementary elective remedy for antibiotic treatments [12,15,19]. Two of the three chosen species (CB and AG) are trees belonging to the Betullacae family and are sometimes used as ornamental plants, while the third belonging to the Glossulariaceae family is mainly used for food. RN, already known by Santa Ildegarda for its healing properties, is the basis of the French liqueur known as Crème di Cassis. The ecology of both AG and RN are adapted in humid climates, while CB grows in shaded areas and requires relatively high temperatures. All three species are native to Europe and they grow in the Italian environments. In addition to these, the C-GM of FC was entered as the control because, according to gemmotherapy protocols, it should not have a strong anti-oxidant, anti-inflammatory and anti-microbial action, while it is prescribed in the treatment of anxiety with gastro-intestinal somatization [12,15,20]. 

In this work, some chemical characteristics of the C-GMs were related to the three main properties that a drug should exhibit in the presence of a microbial infection with cooling symptoms: antioxidant, anti-microbial and anti-inflammatory properties. To the best of our knowledge, only one article studied the chemical composition of GMs [11]. Our study aimed to analyze the chemical composition of the different C-GMs; thus, the concentrations of quercetin and myrecithin, two antioxidant flavonoids abundant in the buds, were evaluated using HPLC analysis. The C-GMs obtained from RN and CB have a higher concentration of these active compounds with respect to AG and FC. Furthermore, HPLC analysis highlights how the intrinsic variation (varieties or chemotypes within a species, and plant ambient conditions) of gems prior to maceration can influence the quality and the concentration of active compounds of C-GMs obtained from the same species with the same extraction method. In particular, data show that the C-GMs from the second producer (B) are the most concentrated. The glucose analysis does not show significant variations between C-GMs obtained from the same species; however, the C-GMs of CB have the highest glucose content, while the other macerates have significantly lower concentrations. Furthermore, the findings obtained by IM-SPME-GC-MS are very considerable because, thanks to this methodology, they highlight for the first time a different volatile chemical composition of the samples analyzed. As shown in the results, components belonging to the terpene family, among which terpinen-4-ol, terpinolene, spathulenol and caryophyllene oxide, were only found in RN extracts. These volatile compounds define the tropism of RN C-GMs for inflammatory diseases of the respiratory tract. Moreover, components belonging to the family of fatty acids have been identified in the C-GMs of FC, CB and AG, even if samples extracted from the same botanical species showed different concentrations between the two sources. Fatty acids are known to be a primary energy substrate, as well as structural components of cell membranes, second messengers, anti-inflammatory and anti-microbial compounds that have beneficial effects for the health of the mother and her offspring, for cardiovascular health, insulin sensitivity, metabolic syndrome, cancer, critically ill patients, and immune system disorders [21,22,23].

It is generally acknowledged that oxidative stress can play a crucial role in the development or chronicity of respiratory tract diseases. In this context, the use of natural products able to neutralize the reactive oxygen species can be a valuable aid in healing [24,25]. The DPPH analysis shows that the B-C-GMs are active at lower concentrations than A-C-GMs, and that C-GMs of CB and AG have the best antioxidant action. Bacterial or viral microbial agents can determine several cooling conditions; among bacterial ones, that associated with *S. pyogenes* is one of the most frequent causes of pharyngitis. The microbial analysis shows that both sources of AG, in equal measure, are the most active C-GMs against this strain as they are able to inhibit bacterial growth at concentrations lower than 2.5% *v*/*v*, and to suppress it at concentrations equal to 5% *v*/*v* and 10% *v*/*v* for AG C-GM from A and B producer, respectively. At these concentrations, this C-GM exerts a selective action towards the pathogenic strain without altering the viability of the probiotic strains. This selective action is already known for natural compounds, and our data support what has recently been observed by Dinu et al. [26]. Therefore, the in vitro data support the integration of ingestible gargles made with C-GMs to treat oral bacterial affections that potentially require the use of antibiotics. Furthermore, a microbial infection induces a natural inflammatory response mediated by the immune system, which is able to counteract the infection but, at the same time, generates important discomforts such as pain, fever and impaired tissue functions. The analyses carried out with the major anti-inflammatory bud-derived GMs, when tested on PBMCs grown in the presence of pro-inflammatory stimulus (LPS), show that the most concentrated extract (RN-B) can significantly counteract the pro-inflammatory response induced by the LPS, bringing the ratio of pro- and anti-inflammatory cytokines to levels close to unstimulated PBMCs (*p* < 0.05). This data supports the traditional use of C-MG of RN as an immunomodulatory agent of the inflammatory response. Finally, the data obtained from the toxicity test indicate that the doses traditionally used (5 gtt of C-GMs in some water/day) have not cytotoxic effects. 

Our in vitro data, in addition to supporting the traditional integration of CB, AG and RN C-GMs in the diet of people with diseases of the upper respiratory tract, highlight how the quality of C-GMs correlates with their activity, and the importance of standardizing all practices carried out before the maceration step in order to obtain more concentrated products which are, therefore, active at lower concentrations.

## 4. Materials and Methods

### 4.1. Glycerine Macerates

The study addressed C-GMs obtained from of buds of FC, CB, AG, and RN from two commercial sources that the Authors decide to obscure since the study has not commercial purposes: A (Naples, Italy) and B (Vielsalm, Belgium) (the identity of the two producers may be disclosed upon specific request).

### 4.2. High Performance Liquid Chromatography (HPLC)

All samples were diluted 1:10 (*v*/*v*) with double distilled water, and reverse-phase chromatography was performed in isocratic conditions, using column C18 (5 µm, 150 × 4.6 mm), conditions 50% H_2_O_2bid_ + 50% acetonitrile as eluent phase, 200 µL of loop volume with 1 mL/min of flow rate and 254 nm wavelength. For this work, a Thermoquest HPLC, Shimadzu (Milano, Italy) was used, with SPD-10A UV-Vis detector and SN4000 controller, Thermo Scientific (Waltham, MA, USA), using Class VP software, Shimadzu (Milano, Italy). Quercetin dihydrate (≥96%, Sigma-Aldrich, St. Louis, MO, USA) and myricetin (≥96%, Sigma-Aldrich, St. Louis, MO, USA) were used as standards.

### 4.3. Solid-Phase Micro Extraction (SPME)

SPME holders and coating fibers were obtained from Supelco (Bellefonte, PA, USA). For SPME sampling, one SPME device (50/30 μm DVB/CAR/PDMS: divinylbenzene/carboxen/polydimethylsiloxane) was used. Prior to use, SPME fiber was conditioned to remove traces of contaminants. Each sample (1 mL) was placed into a 4 mL glass vial, then capped. For IM-SPME, the PDMS fiber was immersed in the sample and held for 30 min at 60 °C. After equilibration, the fiber was inserted into the injection port of the GC–MS system where thermal desorption was performed at 250 °C for two minutes.

### 4.4. Gas Chromatography–Mass Spectrometry

C-GMs were analyzed by GC-MS Perkin Elmer Clarus 500 instrument equipped with a flame ionization detector (FID). Chromatographic separations were performed on a Varian FactorFour VF-1 fused-silica capillary column (length 60 m × 0.32 mm ID × 1.0 μm film thickness). The oven temperature program was as follows: 70 °C, then a gradient of 6 °C/min up to 240 °C, and then a rise to 270 °C at a rate of 6 °C/min to 270 for 20 min. Helium was used as the carrier gas with a flow rate of 1.0 mL/min. The operative conditions of mass spectrometer were: ionization voltage 70 eV; ion source temperature 200 °C; 30.0–500.0 mass range. Mass spectra identification of the volatile compounds was carried out by comparing spectra with those in the NIST02 and Wiley mass spectra libraries. Furthermore, linear retention indices (LRIs) of each compound were calculated using a mixture of n-alkanes hydrocarbons (C8–C30, Ultrasci) injected directly into the GC injector at the same temperature program reported above. The semi-quantitative analysis was performed by normalizing the peak area generated in FID (%) without using corrections factors (RRFs). All analyses were repeated twice.

### 4.5. Electrochemical Measurements

*Glucose analysis*. Glucose analysis through a glucose biosensor, based on the immobilisation of glucose oxidase enzyme on screen-printed electrodes (SPE-PB-GOx) [27] was performed in drop (60 µL) by amperometry, at +50 mV. The unknown samples were analysed with a dilution 1:100 (*v*/*v*) in 0.05 M phosphate buffer + 0.1 M KCl, pH 7.4 (*n* = 3).

### 4.6. 2,2-Diphenyl-1-Picrylhydrazyl (DPPH) Test

The scavenging activities of the C-GMs against DPPH were evaluated. In a 96-well microtiter plate, 20 µL of serial dilutions of GM, starting from 1% *v*/*v* to 0.03% *v*/*v*, were combined with 180 µL of DPPH solution (400 µM). Methanol was used as a negative control. The reaction mixtures were incubated for 30 min at 37 °C in the dark, and the change in absorbance at 517 nm was measured. Mean values were obtained from triplicate experiments. The percentage of radical scavenging activity was calculated using the following equation:

% inhibition = [(A DPPH − A extract)/A DPPH] × 100.

where A DPPH is the absorbance of the DPPH solution only, and an extract is the absorbance of the tested samples. IC_50_ values represent the concentration of organic compounds included in the C-GM in the study at which 50% of DPPH radicals were quenched, and they were expressed as means ± standard deviation for three separate experiments [28].

### 4.7. Microbial Strains

Ten clinical isolates of *Streptococcus pyogenes* derived from pharyngeal swabs, and 10 probiotic strains potentially present in the buccal microbiota were tested. In particular, the *S. pyogenes* isolates were provided by the Dep. of Laboratory and Infectious Science of the A. Gemelli University Hospital Foundation IRCCS of Rome, Italy, while probiotic strains included: two commercial strains of *Lactobacillus acidophilus* LA-14200B and *Lacticaseibacillus casei* [29] RO215; three strains from public collection of *Ligilactobacillus salivarius* subsp. *salivarius* [29] ATCC 11471, *Lacticaseibacillus rhamnosus* (ex *Lactobacillus rhamnosus*) DSM 20021 and *Ligilactobacillus salivarius* subsp. *salicinius* ATCC 11742; five non-commercial strains of *Lactobacillus acidophilus* M141, *Lactiplantibacillus plantarum* [29] M142, *Lacticasebacillus paracasei* subsp. *paracasei* M413, *Bifidobacterium longum* subsp. *longum* B1480, *B. pseudocatenulatum* B669 provided by BUSCOB (Bologna University Scardovi Collection of Bifidobacterium, Bologna, Italy).

### 4.8. Broth Microdilution Susceptibility Testing

Broth microdilution (BMD) susceptibility test according to the European Committee on Antimicrobial Susceptibility Testing [30] international guidelines was performed. Muller Hilton broth (Oxoid, UK) was used to test the antimicrobial activity of all C-GMs against the strains of *S. pyogenes*, while Man, Rogosa, and Sharpe (MRS) broth (Sigma Aldrich, USA) was used to test probiotic strains. The BMD test was performed on a 96-well plate by adding 100 µL of a cell suspension equal to 5 × 10^5^ cfu/mL to a final volume of 200 µL. Scalar dilutions, between 80% *v*/*v* (800 mL/L) and 2.5% *v*/*v* (25 mL/L), of each C-GM were tested. Plates were incubated overnight at 37 °C. After this period, minimum inhibitory concentration (MIC) values were determined by spectrophotometric reading at 450 nm (EL808, Biotek, Winusky, VT, USA). To evaluate the MBC (Minimal Bactericidal Concentration), 5 µL of the content of each well was seeded on standard medium agar plates which were incubated for 24 h at 37 °C. The MIC is defined as the lowest concentration that completely inhibits the organism’s growth compared with the growth in the substance-free control. The MBC is defined as the lowest concentration determining the death of 99.9% or more of the initial inoculum. The lowest inhibitory and bactericidal concentration effective on 90% of the *S. pyogenes* strains were indicated with MIC90 and MBC90, respectively, while the inhibitory and bactericidal concentration for 90% of the probiotic strains were indicated with IC90 and BC90 respectively. Each test was performed in triplicate and in each experiment suitable positive controls and blanks were made.

### 4.9. Isolation of PBMCs

Peripheral blood mononuclear cells (PBMCs) obtained from a buffy coat of 10 healthy donors were isolated with HISTOPAQUE-1077(Sigma Aldrich, USA) solution according to the supplier’s protocols. After collection, 250 µL of a suspension of 1.2 × 10^6^ cells/mL was cultured overnight in sextuplicates at 37 °C in a 48-well plate. PBMCs were incubated in the presence of lipopolysaccharides (LPS) at a concentration of 100 ng/mL alone or in combination with 5% *v*/*v* (50 µL/mL) of the two C-GMs of RN. Untreated PBMC cells were used as control. After 24 h of incubation at 37 °C, the content of 4 replicates was mixed and centrifuged (400 g for 10 min at room temperature) to separate the cells from the culture medium, which were stored at −20 °C until the use. The remaining two replicates were treated with 5 µL of Alamar Blue reagent (Thermo Fisher, Waltham, MA, USA) to ascertain the cellular vitality.

### 4.10. ELISA Assay

The amount of 12 cytokines (IL-1α, IL-1β, IL-2, IL-4, IL-5, IL-6, IL-10, IL-12, IL-13, IL-17A and GM-CSF) involved in the inflammatory response was studied with Multi-Analyte ELISArray kit (MEH-006A. Qiagen, Germany). The test was performed on a 96-well plate; 50 µL cell culture supernatant was diluted 1:2 in each well with 50 µL of dilution buffer and the test was performed according to the Manufacturer’s instructions. Cytokines levels are detected through the OD450 values because, if included in the range identified by the Manufacturer (0.00 and 2.50 units of OD450), they are directly proportional to the real concentration of the cytokines in the sample. For this reason, absorbance values greater than 2.50 and those lower than twice the absorbance values of the negative control were not considered as they are outside the linear range of the assay. For each cytokine, positive and negative controls have been developed.

### 4.11. Cytotoxicity Test

Mouse 3T3 (Swiss albino mouse cell line) fibroblast cell lines were cultured at 37 °C in a humidified environment (CO_2_ 5%) in DMEM with 20 mM Glutamine (Lonza, GmbH, Basel, Switzerland) supplemented with 10% Fetal Calf Serum (Lonza, GmbH), and 500 units/mL Penicillin-Streptomycin-Neomycin Antibiotic Mixture (Lonza, GmbH). 96-well microplates were used to seed 1 × 10^5^ cells in basal medium (200 µL) until a sub-confluent monolayer was reached. After an initial synchronization of the cells grown in a serum free medium for 24 h, the medium was replaced with different solutions of basal medium plus C-GMs in order to achieve the following final percentages: 0.05%, 0.1%, 0.25%, 0.5%, 1% *v*/*v*. Cellular viability was then evaluated at 24 h using the commercial CellTiter-Blue^®^ Cell Viability Assay (Promega, Madison, WI, USA) following the manufacturer instructions. 

### 4.12. Statistical Analysis

Data of the antioxidant activity of the C-GMs was subjected to two-way ANOVA for the factors C-GM plant sources (4 levels), producers (2 levels) and their interaction. Data of inflammatory response (cytokine levels) was subjected to one-way ANOVA for the factor samples (4 levels). To overcome inhomogeneity of variances among ANOVA sources, OD values of the ELISA test were transformed into square root values. In both anti-oxidant activity and inflammatory response, Tukey’s HSD test at *p* < 0.05 was used to separate levels in significant ANOVA sources. The CoStat 6.3 package (CoHort Software, Berkeley, CA, USA) was used for the ANOVA and subsequent Tukey’s test.

## 5. Conclusions

In respiratory diseases, physicians often prescribe locally effective rinses or gargles as a support to conventional therapies. In presence of these diseases, gemmotherapy commonly integrates ingestible rinses or gargles made with 5 drops/day (up to a maximum of 3 times per day) of bud-derived GMs obtained from CB, AG and RN, in order to have both local and systemic activity. Although further evidence is needed, the data obtained in this study support the traditional local use of these bud derivatives, in particular the use of CB for antioxidant activities, and the use of AG and RN as supports for antibiotic and anti-inflammatory therapy, respectively. Furthermore, the study highlights the importance of standardizing cultivation, harvest and processing practices preceding the maceration phase, as well as the need for quality analyses, in order to obtain products that are as concentrated, repeatable, and as effective as possible.

## Figures and Tables

**Table 1:** HPLC analysis of myricetin and quercetin contents in C-GMs.

C-GM	Myricetin (2.7 min)	Quercitin (4 min)	Quercitin (6.3 min)	g/L
FC-A	0.120	0.008	0.359	1.060
FC-B	0.160	n.d.	0.121	1.390
CB-A	0.477	n.d.	n.d	4.230
CB-B	0.566	n.d.	n.d.	5.022
AG-A	0.060	n.d.	n.d.	0.504
AG-B	0.160	0.040	n.d.	1.390
RN-A	0.090	0.020	n.d	0.829
RN-B	0.315	0.024	n.d.	2.781

Note: n.d. = not detected.

**Table 2:** Volatile chemical components (%) of C-GMs.

No. ^a^	COMPONENT ^b^	LRI ^c^	LRI^lit d^	MS ^e^	FC-A	FC-B	CB-A	CB-B	AG-A	AG-B	RN-A	RN-B
1	terpinolene	1073	1079 ^f^	+	-	-	-	-	-	-	8.5	15.8
2	terpinen-4-ol	1165	1168	+	-	-	-	-	-	-	33.1	33.5
3	octanoic acid	1168	1170	+	-	9.9	-	-	-	-	-	-
4	α-methylbenzenepropanamine	1118	1221	+	-	-	-	-	-	-	13.2	11.6
5	dodecanal	1384	1388	+	-	-	-	-	-	3.3	-	-
6	spathulenol	1576	1581	+	-	-	-	-	-	-	15.3	15.9
7	caryophyllene oxide	1580	1585	+	-	-	-	-	-	-	29.8	31.1
8	palmitic acid (C16:0)	1945	1950	+	64.8	31.7	-	20.5	-	31.9	-	-
9	linoleic acid (C18:2)	2128	2130	+	22.8	29.3	-	28.0	-	29.4	-	-
10	linolenic acid (C18:3)	2155	2159	+	12.3	29.0	-	51.4	-	35.4	-	-
	Total identified (%)				99.9	99.0		99.9		100.0	99.9	100.0

Note. ^a^ indicates the compound identification number; ^b^ the components are reported according to their elution order on column; ^c^ Linear Retention Indices measured on apolar column; ^d^ Linear Retention Indices from the literature, identification by MS spectra; -: tr < 0.1%; ^e^ Mass spectrum; +: Comparison with mass spectra reported in Nist library; ^f^ Normal alkane RI.

**Table 3:** The glucose concentration in C-GMs samples.

Sample	Glucose Concentration (Average_mM_ ± St. Dev._mM_)	RSD%
FC-A	0.86 ± 0.06	7
FC-B	4 ± 3	75
AG-A	0.0075 ± 0.0002	3
AG-B	n.d.	-
CB-A	12 ± 1	8
CB-B	15 ± 2	13
RN-A	0.74 ± 0.08	11
RN-B	0.64 ±0.03	5

Note. RSD = Relative Standard Deviation; n.d. = not detectable.

**Table 4:** IC_50_ values of four C-GMs from two different producers (A and B).

	IC_50_ (Average% *v*/*v* ± St. Dev.% *v*/*v*)			
C-GM	FC	CB	AG	RN
A	7.42 ± 1.57 (a)	0.73 ± 0.01 (bc)	1.19 ± 0.28 (bc)	6.67 ± 0.26 (a)
B	2.55 ± 0.49 (b)	0.11 ± 0.00 (c)	0.54 ± 0.01 (bc)	0.62 ± 0.20 (bc)

Note. Different letters (a, b, c) indicate significantly different levels based on the Tukey’s test at *p* < 0.05.

**Table 5:** MIC and MBC values of C-GMs vs. *S. pyogenes* and probiotic strains.

Strain	FC-A	FC-B	CB-A	CB-B	AG-A	AG-B	RN-A	RN-B	FC-A	FC-B	CB-A	CB-B	AG-A	AG-B	RN-A	RN-B
	MIC90 (% *v*/*v*)								MBC90 (% *v*/*v*)							
Sp	10	10	10	5	≤2.5	≤2.5	10	5	80	40	80	40	5	10	80	40
	MIC (% *v*/*v*)								MBC (% *v*/*v*)							
M141	5	5	≤2.5	5	≤2.5	≤2.5	20	≤2.5	40	40	10	10	20	10	40	20
M142	20	10	10	10	5	10	10	5	80	80	80	80	80	40	80	80
M413	10	5	2.5	5	≤2.5	5	10	5	80	80	80	80	40	20	80	40
20021	10	10	10	5	≤2.5	≤2.5	20	10	80	80	80	40	80	80	40	40
11471	≤2.5	20	5	10	≤2.5	≤2.5	5	5	40	40	40	40	80	80	40	20
B1480	80	80	10	10	5	5	10	10	80	80	80	80	40	40	80	80
14200B	20	20	10	20	5	10	10	40	40	20	40	20	20	20	40	20
B669	≤2.5	10	≤2.5	10	≤2.5	≤2.5	≤2.5	≤2.5	20	80	40	40	5	10	20	40
11742	10	10	5	10	5	≤2,5	5	5	10	10	20	10	10	5	10	10
RO215	5	≤2.5	≤2.5	≤2.5	≤2.5	≤2.5	≤2.5	≤2.5	10	20	40	20	5	<2.5	20	<2.5
	IC90 (% *v*/*v*)								BC90 (% *v*/*v*)							
	20	20	10	10	5	10	20	10	80	80	80	80	80	80	80	80

Note. Sp = *S. pyogenes*, M141 = *L. acidophilus*, M413 = *L. paracasei* subsp. *Paracasei*, 20021 = *L. rhamnosus*, 11471 = *L. salivarius* subsp. *Salivarius*, B1480 = *B. longum* subsp. *longum*, 14200B = *L. acidophilus*, B669 = *B. pseudocatenulatum*, 11742 = *L. salivarius* subsp. *salicinius*, RO215 = *L. casei*.

**Table 6:** PBMC cytokines expression in presence of C-GMs and the pro-inflammatory stimulus, and the ratio between the pro-inflammatory cytokines and IL-13.

	Average(OD Value) × 10^−3^									Average Ratio × 10^2^
Sample	IL-1a	IL-2	IL-4	IL-5	IL-10	IL-12	IL-13	IL-17a	GM-CSF	^a^ PRO/IL-13
PBMC	3 ± 1 (b)	24 ± 1	1 ± 0	0 ± 0	2 ± 1 (b)	1 ± 0 (b)	1 ± 0	1 ± 0	3 ± 1 (c)	0.46 ± 0.28 (b)
PBMC + LPS	531 ± 63 (a)	0 ± 4	1 ± 0	0 ± 1	318 ± 26 (a)	6 ± 0 (a)	2 ± 1	1 ± 1	562 ± 21 (b)	7.24 ± 4.00 (a)
LPS + RN-A	537 ± 29 (a)	2 ± 3	2 ± 0	2 ± 1	11 ± 3 (b)	2 ± 0 (ab)	1 ± 0	1 ± 0	1316 ± 129 (a)	18.7 ± 3.30 (a)
LPS + RN-B	47 ± 3 (b)	5 ± 2	2 ± 0	1 ± 1	2 ± 1 (b)	2 ± 1 (ab)	2 ± 0	1 ± 0	39 ± 1 (c)	0.50 ± 0.03 (b)
*p*	<0.01 **	0.12 ns	0.26 ns	0.70 ns	<0.01 **	0.04 *	0.23 ns	0.80 ns	<0.01 **	<0.01 **

Note. ns, * and ** mean not significant and significant at *p* < 0.05 and *p* < 0.01, respectively. Different letters indicate significantly different levels based on the Tukey’s test at *p* < 0.05. ^a^ PRO = Pro-inflammatory cytokines.
